# Color Blindness

**Published:** 1883-12

**Authors:** Julia W. Carpenter


### Color Blindness.

A Paper Read before the Academy of Medicine, Cincinnati, Oct. 1st, 1883.

BY JULIA W. CARPENTER, M.D.

In the latter part of the 18th century there lived the famous English chemist, Dalton. Though a celebrated man, he sometimes, by strange deeds, amazed his friends and caused laughter on the streets. He was a most modest and simple man and a Quaker to whom the color scarlet, is forbidden. Yet when he received, at Oxford, the scarlet gown of a doctor of civil laws, he actually wore it several days in the streets, in utter ignorance of the sensation he created.

When asked the color of his gown, he pointed to some evergreens and said they were alike. The lining of the gown, which was pink silk, was, he said, the same to him as the blue in the sky.

In the description which he gives of his peculiar vision, he remarks among other things that a florid complexion and diluted black ink on white paper had, to him, much the same appearance.

In 1792 he discovered his chromatic defect and studied it with all the energy of a scientist. In 1844 he died after giving to the scientific world, among other things, its chief knowledge of this curious subject. It was two years after the discovery of his peculiarity, that he published the account of his vision and his theory concerning it. This attracted so much attention, that color blindness was afterwards called from him—Daltonism. This was not, however, a correct name, as his was only one form of color blindness, viz., red blindness, and hence the name could not apply to all classes.

Although it is only since his time that color blindness has been known in literature, there is no reason to suppose it has not always existed in man. The first recorded case is that of Harris, the shoemaker. He noticed when a child, that he could not distinguish red fruit, as strawberries, from the leaves around them until near enough to tell them by their form. He found, like others since then, his companions could pick strawberries and cherries more quickly than he. Not being a man of education he made no contribution to the knowledge of this subject, further than to have his own peculiarities recorded.

The mistakes of the color blind are curious and interesting. Some of the reported cases are as follows: With one individual a piece of red sealing-wax thrown on the grass could not be seen, nor a piece of scarlet cloth hung on a green hedge, though others could distinguish it a mile off. The same person saw no difference in the complexion of a foreign lady who had purposely substituted prussian blue for her rouge.

Another called the gray eyes of his sister bluish-red. Another declared a cucumber and a boiled lobster were the same in color with a little difference in shade. Still another could not distinguish between the red berries and green leaves of the mountain ash.

One person discovered his defect in a chemistry class. In an experiment where there was to be a certain change of color, he said he used to stare the whole hour expecting to see it and to him it never occurred. This same individual, when asked to show the color the lips appeared to him, pointed out some green oil paint. He could also see no difference between a blue line of paint on his finger and blood, and was quite annoyed because his companions could. He also bought a purple neck-tie for a black one.

Another, with a taste for drawing, painted a red tree in a landscape. A quaker made some quite conspicuous mistakes. He intended to buy a brown coat for himself, but selected a bottle green, and for his wife, purchased a scarlet merino for some dark shade.

Also a minister selected a scarlet cloth for a new coat, and another man wore a green and a brown glove thinking they were mates.

A post-office clerk in Prussia was always in trouble with his accounts. There was either too little money for the number of stamps sold or too much. At last it was found he could not tell red stamps from green ones, and hence the confusion.

It is supposed in our own country, that many letters find their way to the dead letter office, owing to red two-cent stamps being used instead of the green on account of defect in the color sense.

A particularly interesting case is recorded of one of America's greatest poets. The description given of him says "previous to the time when he discovered his peculiarity, he wondered that people should talk of glorious sunsets and beautiful sunsets as he could neither detect glory nor beauty in them." Yet this person who never saw a glorious sunset, nor the radiant bow of promise, to whom the autumnal foliage, and the hues of the tropics were nothing, wrote by means of his imagination as though he saw it all.

What a vast distance between such a mind and a very ordinary one. One sees nothing and portrays all; the other seeing all may not even appreciate the glowing description.

Color blindness, once only a matter of scientific curiosity, is now known to be of great practical importance to those engaged in the various occupations where it is necessary to have a correct perception of color.

The danger is one of great magnitude when it exists in certain classes of persons. For instance, in the various modes of travel, orders are transmitted by colored signals. Hence the lives of thousands are imperiled if color blindness is found in signal men, engineers, conductors, station-masters, switch-tenders, light-house keepers, and pilots.

European governments have already provided for the exclusion of the color blind from their navies and railroads. The Cunard Steamship Company require the surgeon of the ship to examine the men for this defect. In our country, Massachusetts was the first State to act concerning the control of color blindness on railroads, and every State should follow the example.

Before this subject was understood the mistakes of the color blind seemed without limit; but it is now known, after a close investigation of thousands of cases, that they follow certain fixed laws.

The expression color blind does not mean that one is really blind to colors, that they make no impression on him, but that he sees some of them another way. The color blind themselves are divided into classes, as they do not all see colors the same way. The red blind can no more see as the green blind, than as one of normal vision.

What is called the "Young Helmholtz' Theory of Color Sense" is the one now adopted. It is in brief as follows: There probably are in the eye three sets of nerve elements for the perception of color. Stimulation of one causes the sensation of red; of the second, the sensation of green; of the third, the sensation of violet, the three primary colors. Now, if the elements of the first kind are wanting or paralyzed, there can be no perception of red, if of the second no perception of green, of the third no perception of violet. Accordingly, there is red blindness, green blindness, and violet blindness. The last is quite rare.

Experience, from the examination of thousands of cases corresponds with this theory. These three classes exist, however, in different degrees and with various modifications, so the subject is quite extended in all its details, notwithstanding this classification.

In testing for color blindness, various methods have been used. It has been found that it will not do simply to hold up different colors and ask their names. Knowing the name of a color is a different thing from actually seeing it. The method proved to be the most accurate and simple is that of Holmgren. It is the following: Berlin worsted is the material chosen. It is better for several seasons than bits of colored glass or paper. All the different colors are selected and five shades of each. This pile of worsteds is laid on a table in broad day light. One skein is taken from it and laid by its itself. The one examined is then asked to select from the pile those matching it, and lay them by the other.

One not color blind will pick out quickly all the skeins that are alike, while the others will make the characteristic mistakes. The color chosen as a corresponding one shows the class of color blindness to which he belongs. One is tested in turn for red, green, and violet blindness. When the person examined is one of intelligence, the testing requires no more than a minute.

If one feigned color blindness to escape responsibility for a rail. road accident this examination would quickly reveal the deception. To avoid detection he must be as familiar with the exact laws of the different classes of color blindness as an experienced specialist, which would hardly be probable.

Suppose, for example, the one feigning understood enough of the subject to know there were different classes of this defect, and assumed red blindness, the most common form, and matched red with green or gray, imitating the ordinary mistakes. With a pure red this would be well enough. But show him magenta, a common red. This is not a pure color, but gets its special shade from an admixture of blue. One really red blind does not see the red in it at all, but does see the blue, and that only; hence he matches *this* red with blue. To match it with green or gray, would reveal a deception at once.

Dalton's cloak must have been a pure red, as he compared it to the evergreens. But the silk lining was doubtless a shade of pink, formed by a slight mixture of blue. Hence seeing the blue only, this pink lining was the same to him as the blue in the sky.

One question of great importance in this subject relates to the frequency of its occurrence; and the more accurate and scientific the tests, the greater the number of color blind found. After an examination of thousands of cases, in various countries, by different investigators, it is found that the proportion of color blind among men is one in twenty-five, among women it is very rare indeed.

One European observer, in over one thousand school girls, did not find a single case. Another, in over 2,200 found one. Holmgren himself in over seven thousand women of all ages found nineteen. Dr. Jeffries, of Boston, in nearly eight thousand women, found only four. The proportion of men for this same number would be three hundred and twenty.

As to why color blindness is so common in men and so infrequent in women, no reason has been assigned so we may surmise for ourselves.

Those, not born color blind can, by exercising the eye with colors, cultivate that faculty and by heredity it can be increased. In some of man's early ancestors, according to Darwin, the birds' brilliancy of plumage was given to males only. This, he says, was to make them attractive and win them their mates. Thus the perception of color was cultivated in the female. Not so in the male for he regarded his somber clad companion. His own gray apparel was no cultivation of his own sense of color, for it was given him by nature without any thought of his own.

So then this keen perception of color by females descended, or rose, to the human race.

Now things are reversed, and men wear the somber tints. If, as is often said, women dress, not for each other, but for men, contrary to the birds, will not their color sense in turn be cultivated, so that by the accumulated effect of generations of exercise of this faculty, color blindness may be eliminated from men, and the human race be perfect as to this part of its eye sight.

Color blindness, like other traits, is hereditary and runs in families. In one family it was traced back through five generations, though in some of its branches it skipped over two generations, as occurs with other peculiarities. In this tribe, of 32 men, 18 were color blind; of 29 women but two.

Harris, the shoemaker, had three brothers and Dalton one brother with the same defect.

In most of the families where several brothers had this peculiarity the sisters escaped it altogether. To this rule one strange exception is recorded, in which in four generations of one family those effected were women.

How is it when so many color blind exist, that they pass unnoticed? One reason is the inaccuracy as to the names of colors even among the educated. Some shades hardly any two persons will name the same, but only the one who could not match it with a corresponding shade would be color blind. Dalton, speaking of three of his pupils and himself, said, we thought the trouble was due to a perplexity of names given to particular colors.

The color blind are usually ignorant of their defect until some incident reveals it to them. Mr. Wm. Pole, writing of himself, says it was not until he was thirty years of age that he found out the only two colors he really saw were blue and yellow. All the others, which he had learned by name, appeared to him only as shades of these two.

When different names are given, for what to them are similar colors, as in bricks and grass, cherries and leaves, they look for some finer points of distinction and usually find it in some difference of shade. It is a fact that the color blind have an extraordinary preception of light and shade, and, strange as it may seem, this wonderfully acute perception has even permitted color blind artists to work at their calling, until an accidental substitution of red for green, or vice versa, has revealed the defect.

Is color blindness peculiar to any nation or any special kind of eye? It is found in every European nation and also in the colored race. There is no special color of eye in which it is found. It exists in all, and in about equal numbers. If a German observer reports more blue or gray eyes than black, it is because it is a nation of blondes. If an Italian says black eyes predominate, it is due to the national type being brunette.

Again; color blindness has no connection whatever with errors of refraction, so that whether the eye is near-sighted or the reverse, or has any other departure from the normal type, these defects need not be corrected in testing for color blindness. The eye may be sound and perfect in everything but color. One author reported remarkable visual power as to the shape of objects in many of his cases.

Phrenologists located the faculty of color in the anterior lobes of the brain. Accordingly, we might suppose, the color blind would have a deficiency of that part of the brain, a narrowness of the forehead. One investigator found this true in a majority of his cases. Another, equally patient in measuring, found it greater than usual in the majority of his.

In this subject a great deal of patient toil has been expended with the only result of positively excluding certain points. Many of Nature's truths are reached by the slow process of years of investigation, which result only in exclusion.

The value of negative information was well stated by the famous French savant, who was once asked by a certain lady an apparently simple question in science.

He replied, "Madam, I do not know."

"Well, what is the use of all your scientific education if you cannot tell that?"

"Madam, to be able to say I do not know."

What is the cause of color blindness? One is born so, and can not be educated out of it any more than out of any other congenital defect. It cannot be cured by exercise with colors, nor by any means now known. Whether the defect is in the brain or the retina, is a question not yet settled.

Can color blindness be acquired? The color sense can be weakened or perverted by injuries, some diseases, and by the use of tobacco and alcohol, and this not an excessive use.

The accidents, which can induce this result, are those that cause great jars and shocks, as railroad accidents. One interesting case is reported of a physician, who was thrown from his horse and suffered from it great cerebral disturbance. After recovery from this, on going into his garden, he was greatly shocked to find his beautiful damask rose--flower, stem, and leaves--all of one dull, uniform color, nor did they ever regain for him their original tints.

The diseases that cause color blindness are some affections of the eye or brain and nervous system, and when the malady itself is cured the chromatic defect disappears.

A curious case is related by Tyndall, where color blindness followed straining of the eyes with needlework. A sea captain used to while away some leisure hours by embroidering. One afternoon he worked until twilight. Then, not being able to see the colors distinctly, he changed his seat for a better light, and continued his work, when suddenly his vision for colors vanished, and never returned to him. Two bundles of worsteds shown him —one red, the other green—were both drab to him with only a difference of shade.

Warning: Not too much Kensington.

Of cases of acquired color blindness those of the most interest and greatest frequency are caused by tobacco and alcoholic beverages, especially the latter. At one of the large eye clinics in Paris scarcely a day passed without examples of this kind appearing, and one learns to diagnose them very quickly without any other effect of alcohol being apparent.

The patient usually presents himself with great loss of vision. No glasses relieve the difficulty. The chief means of diagnosis is the color test. The one franc silver piece and the twenty franc gold piece, being about the same in size, they were usually shown one or the other of these and asked which it was. They would pick it up, scrutinize it, and finally say they could not see whether it was gold or silver.

Sometimes one of these patients would come in greatly alarmed saying he had just discovered in making change he could not tell these coins apart, and hence he hastened to an occulist.

When told the cause of the trouble they were usually at first incredulous, as many of them were only moderate drinkers, so-called, but moderation was too much for them. There was but one remedy —immediate and absolute total abstinence. When told this, their frequent reply was, " C'est raide ; " ( " It is rough," ) expressing their almost despair, and it was sad to see how severe the remedy appeared to them. When the advice was followed the trouble disappeared when it had not been of too long standing.

In going over the different phases of this subject, we pass through the various shades of feeling from gay to grave, from the laughable, harmless blunders to blighted lives. It is a fruitful theme and suggests many illustrations for moral truths.

Dr. Oliver Wendell Holmes thus teaches the lesson of charitableness to each other :

" Why should we look one common faith to find,
Where one in every score is color blind,
If here on earth they know not red from green,
Will they see better into things unseen ? "

—*Lancet and Clinic.*

